# Critical Role of Interferon-α Constitutively Produced in Human Hepatocytes in Response to RNA Virus Infection

**DOI:** 10.1371/journal.pone.0089869

**Published:** 2014-02-26

**Authors:** Yoji Tsugawa, Hiroki Kato, Takashi Fujita, Kunitada Shimotohno, Makoto Hijikata

**Affiliations:** 1 Laboratory of Human Tumor Viruses, Institute for Virus Research, Kyoto University, Kyoto, Japan; 2 Laboratory of Viral Oncology, Graduate School of Biostudies, Kyoto University, Kyoto, Japan; 3 Laboratory of Molecular Genetics, Institute for Virus Research, Kyoto University, Kyoto, Japan; 4 The Research Center for Hepatitis and Immunology, National Center for Global Health and Medicine, Ichikawa, Japan; Kobe University, Japan

## Abstract

Several viruses are known to infect human liver and cause the hepatitis, but the interferon (IFN) response, a first-line defense against viral infection, of virus-infected hepatocytes is not clearly defined yet. We investigated innate immune system against RNA viral infection in immortalized human hepatocytes (HuS-E/2 cells), as the cells showed similar early innate immune responses to primary human hepatocytes (PHH). The low-level constitutive expression of IFN-α1 gene, but not IFN-β and IFN-λ, was observed in both PHH and HuS-E/2 cells in the absence of viral infection, suggesting a particular subtype(s) of IFN-α is constitutively produced in human hepatocytes. To examine the functional role of such IFN-α in the antiviral response, the expression profiles of innate immune-related genes were studied in the cells with the treatment of neutralization against type I IFN receptor 2 (IFNAR2) or IFN-α itself to inhibit the constitutive IFN-α signaling before and after virus infection. As the results, a clear reduction of basal level expression of IFN-inducible genes was observed in uninfected cells. When the effect of the inhibition on the cells infected with hepatitis C virus (HCV) was examined, the significant decrease of IFN stimulated gene expression and the enhancement of initial HCV replication were observed, suggesting that the steady-state production of IFN-α plays a role in amplification of antiviral responses to control the spread of RNA viral infection in human hepatocytes.

## Introduction

It has been shown that the replication of RNA viruses, including Sendai virus (SeV), Vesicular stomatitis virus, Newcastle disease virus, Sindbis virus, and Hepatitis C virus (HCV) is suppressed by type I interferon (IFN) (IFN-α and IFN-β) produced rapidly from the cells after infection of viruses [1], [2]. The cytosolic RNA helicases, including retinoic acid-inducible gene (RIG)-I, have been identified as receptors of pathogen-associated molecular patterns of RNA viruses [3]. After recognition of RNA virus infection by those receptors, signal cascades for induction of IFNs are stimulated and result in the activation of IFN regulatory factor (IRF)-3, and IRF-7 which are transcription factors for induction of type I IFN genes [3], [4]. IRF-3 is constitutively produced in many cell types and tissues and phosphorylated after virus infection. Phosphorylated IRF-3 forms a dimer (either a homodimer or a heterodimer with IRF-7) and is translocated to the nucleus. Unlike IRF-3, IRF-7 is constitutively produced only in small amounts, if any, but the gene expression is strongly induced by type I IFN-mediated signaling in most cell types and tissues. The produced IFNs by the way described above then are secreted from the viral infected cells and bind to their receptors, which consist of IFN α/β receptor (IFNAR1 and 2), on the surface of IFN producing and/or neighboring cells and deliver the IFN signal to those cells. The primary role of type I IFN is to limit spread of a variety of pathogens via initiation of the innate immune responses through induction of the genes encoding cytokines and antiviral proteins during the first days of infection [5]. These proteins exhibit antiviral effects both directly by inhibiting viral replication and indirectly by stimulating the adaptive immune system.

On the other hand, constitutive production of type I IFN has been detected in several cells without pathogen stimulation albeit at low level. The constitutive IFN-β has been revealed to be important in maintaining immune homeostasis and essential for immune cell priming [6]. In addition, type I IFN is also known to correlate with multiple biological activities, including anti-proliferative, anti-tumor, pro-apoptotic, and immunomodulatory functions [6], [7]. Previously it was reported that the IFN-α mRNA was expressed in the human normal liver [8]. However, what cell type in the human liver is responsible for the expression and what is the biological role have not been clear yet.

Our previous data showed that immortalized human hepatocytes, HuS-E/2 cells, support the whole life cycle of blood-borne HCV (HCVbb) [9]. The infection and proliferation of HCVbb in HuS-E/2 cells were enhanced by ectopic expression of a dominant-negative form of IRF-7, but not that of IRF-3, suggesting that IRF-7, rather than IRF-3, plays a primary role in regulation of HCV proliferation in these cells [10]. IRF-7 mRNA was detected in primary hepatocytes (PHH) and HuS-E/2 cells, but not HuH-7 cells, one of hepatocellular carcinoma derived cell lines, without virus infection [10]. However, the role of the preexisting IRF-7 and molecular mechanism of its constitutive expression in hepatocytes remains unclear, because of limited knowledge of immediate innate immune response in human hepatocytes.

As the result of study on innate immunity of human hepatocytes, here we report that active IFN-α release occurs in human hepatocytes even in the absence of virus infection. We additionally show that the constitutive IFN-α plays a critical role in the early induction of IFN genes and some IFN stimulated genes (ISGs) through the increase in expression of genes related with induction of such genes, including IRF-7 gene, before virus infection.

## Materials and Methods

### Cell Culture

HuH-7, Huh-7.5, HepG2, and 293FT cells were grown in Dulbecco’s modified Eagle’s medium (Nacalai Tesque, Kyoto, Japan) supplemented with 10% fetal bovine serum, 100 U/ml nonessential amino acids (Nacalai Tesque, Kyoto, Japan), and Antimycotic Mixed Stock Solution Penicillin 100 units/ml, Streptomycin 100 µg/ml, Amphotericin B 0.25 µg/ml (Nacalai Tesque, Kyoto, Japan). HuS-E/2 cells were cultured as previously described [10]. PHH were purchased from Gibco (Grand Island, NY, USA), the hepatocyte donor was a 58-year-old male who did not show any evidences of liver abnormalities. PHH were cultured in serum-free hepatocyte maintenance medium (Gibco, NY, USA) for one week before starting each experiment.

### Treatment with Neutralizing Antibodies (nAb) and Recombinant IFN

Two days prior to stimulation or infection, HuS-E/2 cells were seeded on the collagen coated 12 well plate (8×10^4^ cells/well) to yield a confluent cell layer within 24 h. In the case of infection experiment, the cells were treated with nAb mentioned below for 12 hours (hrs). Then the cultured medium contained nAb were replaced with new culture medium containing Sendai virus (SeV) or cell culture derived recombinant HCV (HCVcc) after wash with phosphate buffer saline (PBS). SeV was prepared as described previously [10]. HCVcc was prepared from the HuH-7 or Huh-7.5 cells transfected with in vitro synthesized Jikei Fulminant Hepatitis (JFH) 1^E2FL^ RNA as described previously [11]. Recombinant human IFN-α was obtained from Merck (Darmstadt, Germany). Blocking Antibodies targeting IFN-α (MMHA-2), IFN-β (Rabbit polyclonal antibody), and IFNAR2 (MMHAR-2) were purchased from PBL Biomedical Laboratories (Piscataway, NJ).

### RNA Extraction, Reverse-transcriptase Polymerase Chain Reaction (RT-PCR), and Quantitative RT-PCR (qRT-PCR)

Total RNA was isolated from cell cultures with Qiagen RNeasy Mini Kit (Qiagen GmbH, Hilden, Germany). Using 200 ng of total RNA as a template, RT-PCR was done with one-step RNA PCR kit (Takara, Kyoto, Japan) according to the manufacturer’s instruction. qRT-PCR was performed with One Step SYBR PrimeScript PLUS RT-PCR Kit (Takara, Kyoto, Japan) by using 7500 Real-time PCR system (Applied Biosystems, Carlsbad CA) according to the manufacturer’s instruction. The primer sets used in those PCRs are detailed in Table S1. Real-time PCR data are given as the mean of triplicate samples with standard deviation. The value obtained for the untreated control sample was generally set to 1.

### Primer Design and Selection

The primers were designed based on the conserved specific sequence of IFN genes, using primer design software Primer-BLAST (National Center for Biotechnology Information. USA).

To evaluate the sensitivity and specificity of designed primer sets, RT-PCR using those primer sets and in vitro synthesized RNAs for subtypes of IFN as templates was performed. To make the in vitro expression plasmids for the IFNs, the cDNA fragment of each subtype of IFN was synthesized from total RNA from IFN-α stimulated HuS-E/2 cells by RT-PCR and subcloned in to the multiclonning sites of pcDNA3. The RNA fragment of each IFN subtype was synthesized with the plasmid in vitro using the MEGAscript T7 kit (Ambion, Austin, TX) according to the manufacturer’s protocol. Synthesized RNA was treated with DNase I followed by acid phenol extraction to remove any remaining template DNA and used for RT-PCR by using one-step RNA PCR kit (Takara, Kyoto, Japan). The amplification conditions were 2 min preheating at 94°C, followed by from 25 to 35 cycles of 10 sec denaturation at 94°C, 30 sec annealing at 55°C, and 1 min elongation at 72°C.

### Quantification of Type I IFN

Quantification of active type I IFN was performed by HEK-Blue IFN-α/β cells (Invivogen, San Diego, CA) according to the manufacturer’s instruction. Type I IFN concentration (U/ml) was extrapolated from the linear range of a standard curve generated using known amounts of recombinant IFN-α (Merck Darmstadt, Germany).

### Immunoblotting

Immunoblotting analysis was performed essentially as described previously [12], with slight modifications. Samples of cell lysates were prepared in lysis buffer (50 mM Tris-HCl (pH 8.0), 0.1% SDS, 0.5% sodium deoxycholate, 150 mM NaCl, 5 mM EDTA, 1 mM Orth vanadate (sigma-aldrich, St.Louis, USA), 10 mM NaF, Protease Inhibitor Cocktail (sigma-aldrich St.Louis, USA)). Antibodies used in this study were polyclonal rabbit antiserum against RIG-I at a 1∶1000 dilution, IRF-7 at a 1∶1000 dilution, STAT1 at a 1∶1000 dilution, or p-STAT1 at a 1∶1000 dilution. These antibodies were purchased from Cell Signaling Technology (Beverly, MA, USA). Immune-complexes were detected using Western Lightning reagent (PerkinElmer, Waltham, MA) by LAS-4000 system (Fujifilm, Tokyo, Japan).

### ELISA for Human IFN-α Protein

Conditioned medium from culture system of HuS-E/2 cells infected with SeV, was collected at 3 and 12 hrs post-infection. The concentration of IFN-α in the conditioned medium was measured with human IFN-α enzyme-linked immunosorbent assay (ELISA) kit (PBL Biomedical Laboratories).

### Indirect Immunofluorescence Analysis

Indirect immunofluorescence (IF) analysis was performed essentially as described previously [11]. The primary antibody was anti-SeV polyclonal antibody (1∶200) (MBL International Corporation, MA, USA). The fluorescent secondary antibody was Alexa 568-conjugated anti-rabbit (Invitrogen, Carlsbad, CA). Nuclei were stained with 4′,6-diamidino-2-phenylindole (DAPI). The fluorescent signals were visualized by fluorescence microscopy (Bio Zero Keyence Co.).

## Results

### Induction of IFN Genes and ISGs in PHH and HuS-E/2 Cells by the Infection of Sendai Virus

**Figure 1:**
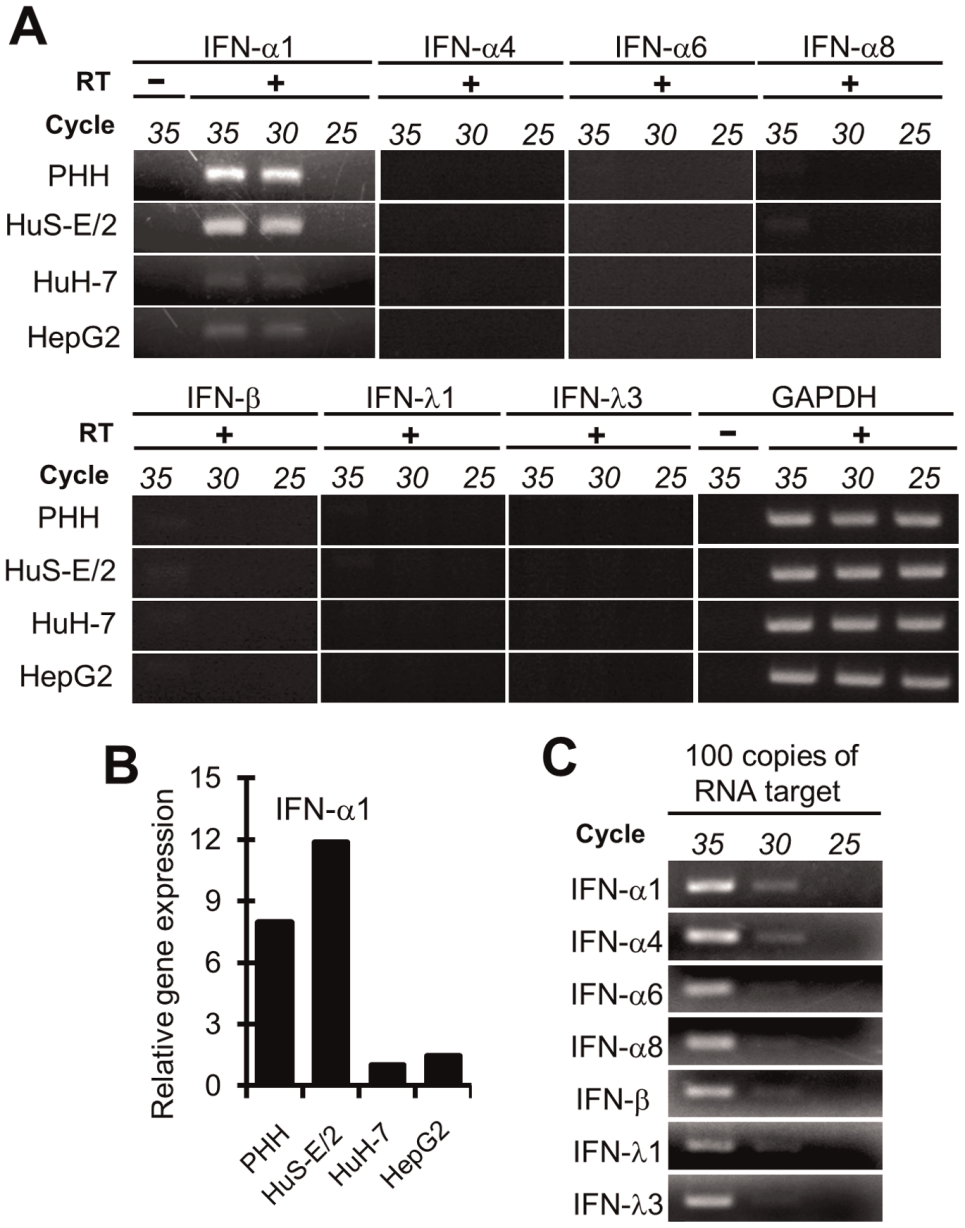
The expression of IFN-α1 gene in the cells derived from human hepatocytes. *A,* The expressions of several IFN genes in human hepatocyte derived cells. The presence of mRNAs for several IFN genes, IFN-α1, IFN-α4, IFN-α6, IFN-α8, IFN-β, IFN-λ1, and IFN-λ3, in PHH, HuS-E/2, HuH-7, and HepG2 cells without virus infection was examined by RT-PCR with different amplification cycles, 25, 30 and 35 cycles. RT-PCR was done with (+) or without (−) reverse transcriptase (RT) reaction. PCR products were separated in the agarose gel and stained with ethidium bromide. GAPDH mRNA was used as an internal standard substance. *B,* Semi-quantitative estimation of IFN-α1 mRNA in those cells. The relative amount of mRNA for IFN-α1 in those cells was estimated by qRT-PCR. The results of qRT-PCR were presented as relative gene expression using the RNA level of HuH-7 cells as a benchmark. *C*. Evaluation of sensitivity of RT-PCR system employed in the detection of IFN subtypes in small quantity. To show the sensitivity of RT-PCR system in this study, RT-PCR was performed with different amplification cycles, 25, 30 and 35 cycles using a primer set described in Table S1 and one hundred copies of in vitro synthesized RNA of each IFN subtype as a template.

We examined the antiviral responses of IFN system in some human hepatocyte derived cells against RNA viral infection using sendai virus (SeV) which is a negative strand RNA virus and has been widely used in studies on induction of IFN system [13]. The constitutive expression of IRF-3 and RIG-I genes was commonly found in all those cells as already repoted (data not shown, [10]). Our previous observation that IRF-7 gene was expressed in PHH and HuS-E/2 cells but not in HuH-7 cells under this condition was also confirmed (data not shown, [10]). In addition, the expression of IFN-α1 gene was newly observed in PHH and HuS-E/2 cells albeit at low level (Fig. 1*A*). That, however, was not in the case in HuH-7 and HepG2 cells, although the RT-PCR product was sometimes seen in HuH-7 and HepG2 cells but only vaguely (Fig.1*A*). These results were also confirmed quantitatively by qRT-PCR (Fig. 1*B*). In order to examine the gene expression of the other IFN subtypes in those cells, the primer sets for RT-PCR to detect mRNAs for those factors sensitively and specifically were designed and obtained to use for specific amplification of about one hundred copies of IFN-α4, IFN-α6, IFN-α8, IFN-β, IFN-λ1, or IFN-λ3 mRNAs (Fig. 1*C* and Table S1). Those mRNAs, however, were not detected at all in all cells used in this study by this RT-PCR condition (Fig. 1*A*). These results suggested that IFN-α, at least IFN-α1, but not IFN-β and IFN–λ, is slightly produced in human hepatocytes without virus infection.

**Figure 2:**
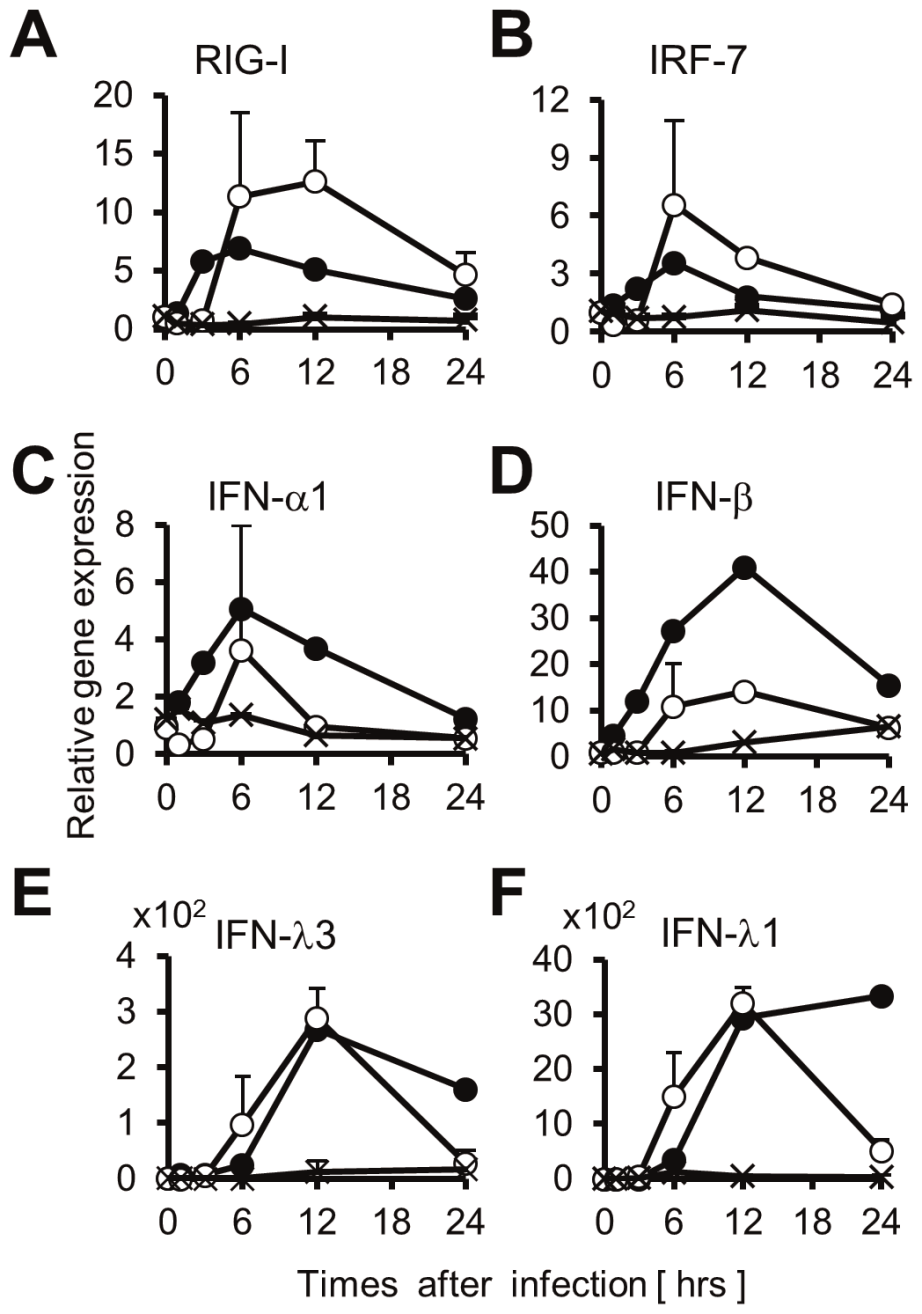
Expression profiles of genes associated with IFN signals in the cells after infection of SeV. Total RNAs were purified from PHH (closed circles), HuS-E/2 cells (open circles), and HuH-7 cells (cross marks) at indicated time points after infection of SeV. Relative quantities of RIG-I (*A*), IRF-7 (*B*), IFN-α1 (*C*), IFN-β (*D*), IFN-λ3 (*E*), and IFN-λ1 (*F*) mRNAs in each total RNA sample were examined by qRT-PCR and plotted using the RNA level firstly detected as a benchmark. The quantity of each RNA sample was normalized by the amount of GAPDH mRNA as relative gene expression. Error bars represent standard deviation (SD) of the mean of determinations from three experiments.

Next, the effect of SeV infection on mRNA levels of those genes was investigated in those cells. The mRNA levels of IRF-7 and IFN-α1, a target gene product of IRF-7, were transiently increased and reached peaks 6 hrs post-infection (p.i.) in both PHH and HuS-E/2 cells (Fig. 2*B and 2C*, closed and open circles). Those of IFN-β, and IFN-λ3 reached peaks 12 hrs p.i. in those cells in similar ways (Fig. 2*D and 2E*, open and closed circles). IFN-λ1 mRNA was also increased by 12 hrs p.i. in both HuS-E/2 cells and PHH (Fig. 2*F*, open and closed circles). It decreased from 12 to 24 hrs p.i. in HuS-E/2 cells (Fig. 2*F*, open circles) whereas it slightly increased in PHH (Fig. 2*F*, closed circles). The gene expression of RIG-I, a key factor in the innate immune response to RNA virus infection in many cells [14], [15], was also investigated. The increase of RIG-I mRNA was observed from 1 hr to 6 hrs p.i. in PHH (Fig. 2*A*, closed circles). Although that was observed from 3 hrs to 12 hrs p.i., the similar pattern of the increase was observed in HuS-E/2 cells (Fig. 2*A*, open circles). The expressions of RIG-I, IRF-7, and IFNs genes were also assessed in HuH-7 cells following infection. Obvious increase of those mRNAs, however, was not detected in telling contrast to PHH and HuS-E/2 cells (Fig. 2, crosses). These observations indicated that this early innate immune response to SeV infection did not occur in HuH-7 cells. The absence of this early response may explain why HuH-7 cells support efficient proliferation of recombinant HCV [16]. On the other hand, the innate immune response of HuS-E/2 cells against SeV infection seemed to be relatively similar to that of PHH, although the responsiveness curves of those gene expression were slightly different. Thus, we supporsed that HuS-E/2 cells provide a valuable model for studying innate immune response of human hepatocytes against viral infection.

### Active IFN-α was Constitutively Produced in HuS-E/2 Cells at a Low Level

In this study, we addressed the constitutive expression of IFN-α1 gene observed in PHH and HuS-E/2 cells, since the detection of IFN-α1 mRNA in the liver tissues from normal human individuals was already detected by RNA blot hybridization, but its function was not cleared yet [8].

**Figure 3:**
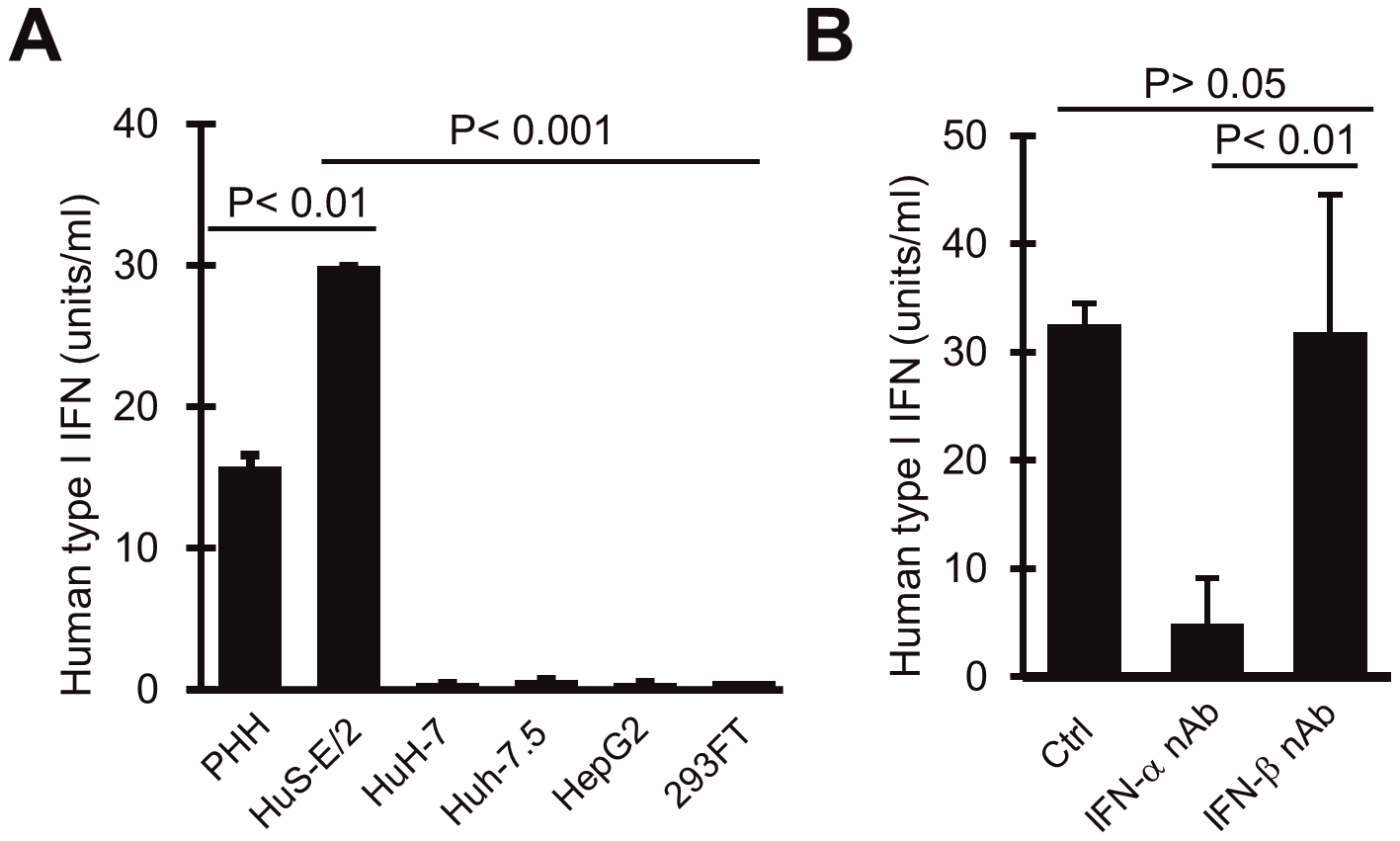
Activity of IFN-α constitutively produced into the culture medium. *A*, Activity of type I IFN produced from the cells without virus infection. Activities of type I IFN produced from PHH, HuS-E/2, HuH-7, Huh-7.5, HepG2, and 293FT cells in the culture media were examined by HEK-Blue™ IFN-α/β cells as described in experimental procedures section and plotted in the graph. *B,* Production of IFN-α from the cells without virus infection. Activity of type I IFN produced from HuS-E/2cells in the culture medium over night was examined as *A*, after treatment with 5 µg/ml neutralizing antibody (nAb) against IFN-α (IFN-α nAb) or IFN-β (IFN-β nAb) for 2 hrs. Error bars represent SD calculated from results of three independent experiments. Probability value (*P* value) was calculated with Student’s t test.

The presence of IFN-α in the conditioned medium from HuS-E/2 cell culture system was examined firstly by the ELISA system for IFN-α. However, that was not detected probably because of low concentration of secreted IFN-α and low sensitivity of the system. Then the activity of type I IFN in the culture medium assessed by using HEK-Blue type I IFN assay system in which the cells are designed to produce embryonic alkaline phosphatase protein in type I IFN receptor signaling-dependent manner. As shown in Fig. 3*A*, the culture medium from HuS-E/2 cell culture system showed significantly higher activity of type I IFN than those from the culture systems for Huh-7.5, HuH-7, and HepG2 cells as well as 293FT cells, a cell line derived from human embryonic kidney. To confirm above results further, the effect of neutralizing antibody (nAb) targeting IFN-α on the above conditioned medium was examined similarly. As shown in Fig. 3*B*, the pretreatment of the conditioned medium from HuS-E/2 cell culture with this antibody effectively suppressed the activity of IFN receptor signaling, while nAb targeting IFN-β did not affected (Fig.3*B*). These observations indicated that functional type I IFN was included in the conditioned medium from HuS-E/2 cell culture system and, at least, the majority of the type I IFN in the medium was IFN-α and not IFN-β. From above results, we concluded that HuS-E/2 cells produce functional IFN-α into the culture medium without virus infection.

### The Basal Expression of IFN-α1 and ISGs was Elevated by Type I IFN Receptor Signaling

**Figure 4:**
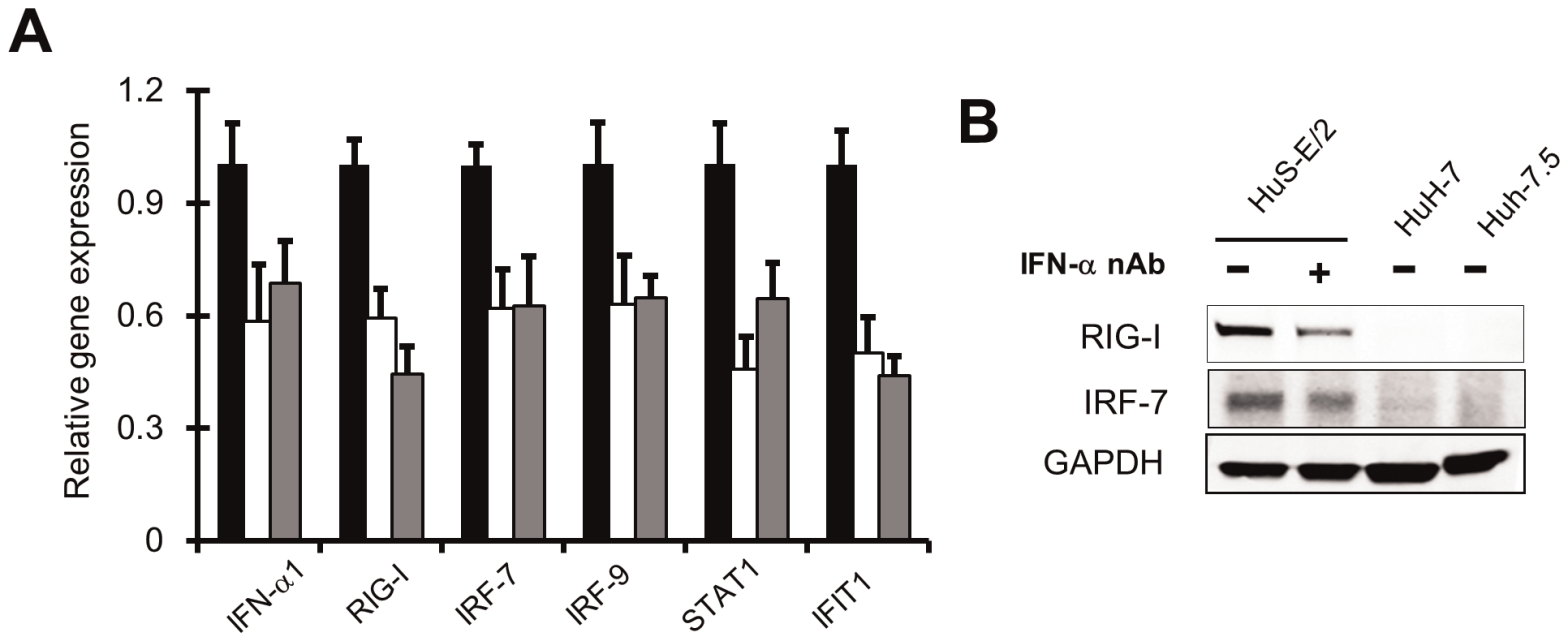
The function of constitutive IFN-α1 on the expression of the genes associated with IFN signals. *A,* The mRNA levels of the genes associated with IFN signals, RIG-I, IRF-7, IRF-9, STAT1, IFIT1, and IFN-α1 itself were examined in HuS-E/2 cells treated with and without nAb against IFN-α (IFN-α nAb, gray bars) (5 µg/ml) or IFNAR2 (IFNAR2 nAb, white bars) (5 µg/ml) for 12 hrs by qRT-PCR. Relative expression level of those genes are plotted using the RNA levels detected in the cells treated without nAb (Mock, black bars) as a benchmark. Results were derived from three independent experiments and error bars represent the calculated SD. *B,* Total cell lysates of HuS-E/2 cells treated with (+) or without (−) 5 µg/ml IFN-α nAb for 12 hrs were analyzed with immunoblotting using antibodies against RIG-I, and IRF-7. Those of HuH-7 and Huh-7.5 cells without treatment were also investigated. Protein levels was normalized among the samples by levels of GAPDH detected with anti-GAPDH antibody.

To see whether the IFN-α1 produced in the cells without virus infection affects the cells through the autocrine or paracrine signaling regardless of low level production, the basal expression of several ISGs in HuS-E/2 cells cultured in the medium containing nAb targeting IFNα/β receptor (IFNAR) 2, one of the receptors for type I IFN, were investigated. As shown in Fig. 4*A*, it was clearly observed that the mRNA levels of RIG-I, IRF-7, IRF-9, signal transducer and activator of transcription (STAT) 1, and IFN-induced protein with Tetratricopeptide 1 (IFIT1) were diminished in the cells with the nAb treatment, compared to mock treated cells. Quite similar results were observed in the cells treated with nAb targeting IFN-α (data not shown). The basal expression level of IFN-α1 gene was also reduced by these treatments (Fig. 4*A*). Decreased protein levels of RIG-I and IRF-7 were also observed in HuS-E/2 cells treated with nAb targeting IFN-α, while these proteins were below the detectable level in HuH-7 and Huh-7.5 cells as predicted (Fig. 4*B*). As the treatments of two different nAbs showed the similar results to each other, it was suggested that the type I IFN receptor signaling upregulates the expression of those ISGs, as well as IFN-α1 gene, in basal level in HuS-E/2 cells to some extent.

### Basal Production of IFN-α in HuS-E/2 Cells Contributes to Rapid Antiviral Response during Early Phase of Infection

**Figure 5:**
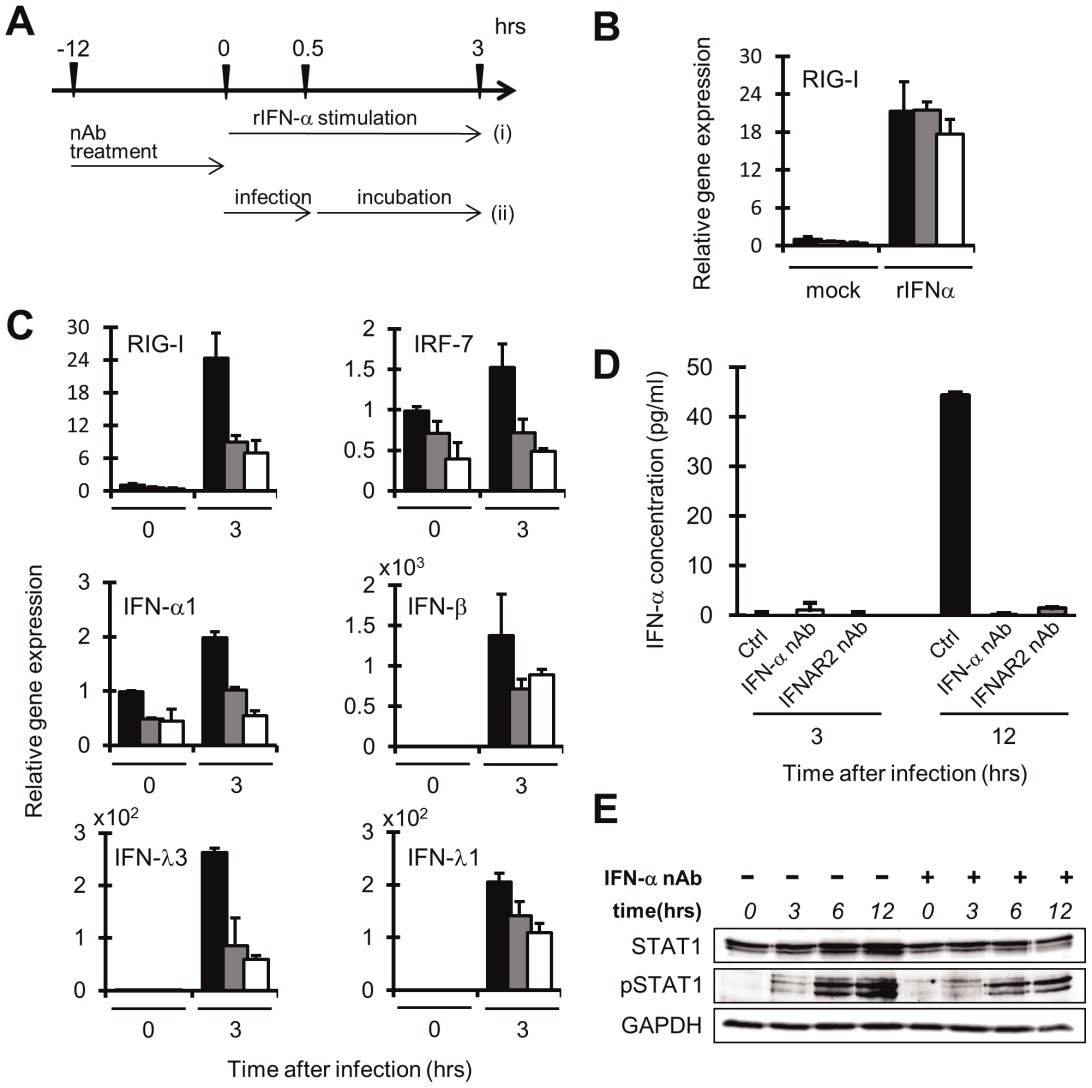
Roles of Constitutive IFN-α on the virus-induced antiviral responses in HuS-E/2 cells. *A*, Schematic of the time schedules for the experiments of recombinant human IFN-α (rIFN-α treatment (i) or SeV infection (ii) after nAb treatment. *B*, Responses of the cells pretreated with IFN-α or IFNAR2 nAbs against exogenous stimulation of rIFN-α. RIG-I mRNA in the cells treated with rIFN-α (2.5 unit/ml) (i) for 0 and 3 hrs after the treatment with IFNAR2 nAb (gray bars), IFN-α nAb (white bars) or mock (black bars) were analyzed by qRT-PCR. Relative expression level of those genes are plotted using each RNA level detected in the cells treated with mock for 0 hr as a benchmark (*B*, *C*). Error bars represent the calculated SD from the results obtained in three independent experiments (*B*, *C*, *D*). *C,* Responses of the cells pretreated with IFN-α or IFNAR2 nAbs against SeV infection. IFN-α1, IFN-β, IFN-λ1, IFN-λ3, and IRF-7 mRNAs in the cells processed as described for panel *B* except infection of SeV (ii) instead of rIFN-α treatment, were analyzed by qRT-PCR. *D*, SeV infection induced IFN-α production from the cells pretreated with IFN-α or IFNAR2 nAbs. Quantity of IFN-α protein in the culture medium was determined by ELISA at 3 and 12 hrs post-infection of SeV. The cells were processed as described for panels *C. E*. STAT1 phosphorylation in the cells pretreated with IFN-α nAbs after SeV infection. The phosphorylation status of STAT1 in the cells processed as described for panel *D* except pretreatment with (+) or without (−) IFN-α nAb only was analyzed by western blot analysis using anti-STAT1 antibody (STAT1), anti-phosphorylated STAT1 antibody (pSTAT1) at 3, 6, and 12 hrs post-infection of SeV. Protein levels were normalized among the samples by levels of GAPDH detected with anti-GAPDH antibody.

To study the functional role of basal level production of IFN-α in HuS-E/2 cells, the innate immune responses of the cells treated with the above nAbs against the RNA virus infection was investigated. In this experiment, HuS-E/2 cells pretreated with nAbs for 12 hrs to interrupt the basal IFN signaling were exposed to the fresh culture medium contained SeV for 30 min after rinsing the nAbs off from the cells. After the infection, the cells were cultured for 3 hrs and then used as follows (Fig. 5*A*). As the induced expression of RIG-I gene, used as a representative of ISGs, by the treatment with recombinant IFN-α was observed similarly in both cells pretreated with and without nAbs (Fig. 5*B*), it was clearly shown that the cells pretreated with the nAbs retained responsiveness to IFN-α rinsing procedure. At first, the levels of mRNAs for RIG-I, IRF-7, IFN-α1, IFN-β, IFN-λ1, and IFN-λ3 were examined in the cells with or without SeV infection. As shown in Fig. 5*C*, almost the similar patterns of increases of those mRNAs were observed in the cells with SeV infection, although only minor levels of the induction were found in the cases of IRF-7 and IFN-α mRNAs even in the mock pretreated cells. In all the cases, nAb pretreatments effectively suppressed the increases of those mRNAs 3 hrs after SeV infection (Fig. 5*C*). As shown in Fig. 5*D*, the suppression of IFN-α protein production in the culture medium 12 hrs after the infection was also observed by using ELISA.

Next, to examine the effect of the pretreatments with nAbs on virus-induced STAT1 activation, phosphorylated form of STAT1, activated form of STAT1, was detected in SeV infected cells pretreated with nAbs by western blot analysis using anti-phosphorylated STAT1 (pSTAT1). As shown in Fig. 5*E*, the level of pSTAT1 found in the SeV infected cells without IFN-α nAb pretreatment was apparently reduced in the cells with pretreatment at 3, 6 and 12 hrs post-infection, although the protein levels of STAT1 were not affected by the pretreatment, suggesting that pretreatment by IFN-α nAb suppressed the IFN signaling induced by virus infection.

These results suggested that IFN-α produced in the HuS-E/2 cells without virus infection plays a role in the enhancement of initial response of IFN system.

### IFN-α Released from HuS-E/2 cells without Viral Infection Contributes to Inhibit Initial Infection and Proliferation of RNA Viruses, Including HCV

**Figure 6:**
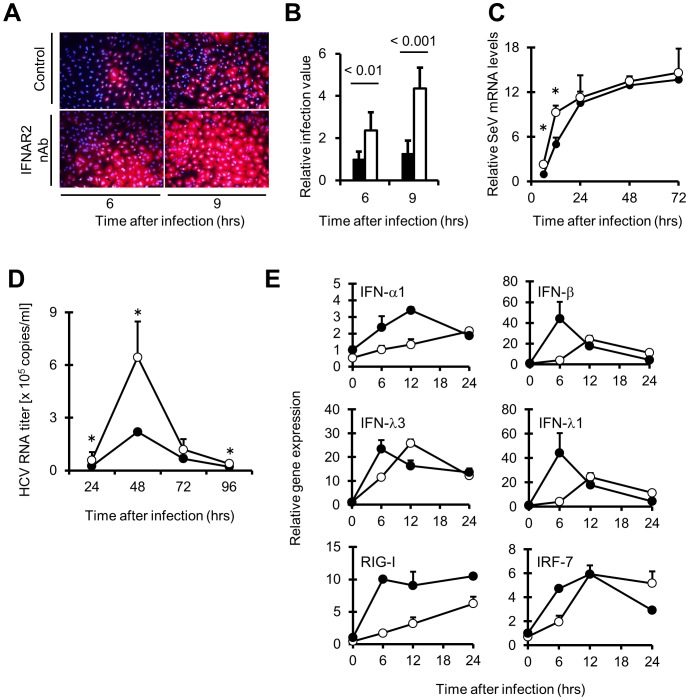
Suppressive role of constitutive IFN-α on viral infection or initial viral proliferation. *A*, Enhanced infection of SeV by neutralization of constitutive IFN-α visualized with IF. Cells were processed basically as described in the figure legend for Fig. 5, panel *C* (*A, B, C, D, E*). At 6 and 9 hrs after SeV infection, the cells were fixed and studied by IF using anti SeV antibodies (red). Nuclei were stained with DAPI (blue). *B*, Numerical conversion of the results in panel *A*. Infection of SeV was quantified by counting of the fluorescent positive cells in ten fields of views per well of two wells. Relative infection values of the cells treated with (white bars) and without (black bars) IFNAR2 nAb were calculated by using the averaged number of infected cells without nAb treatment at 6 hrs postinfection as a benchmark. *P* value was calculated with Student’s t test. *C*, Quantification of SeV RNA were measured by qRT-PCR. RNA samples from HuS-E/2 cells pre-treated with (open circles) and without (closed circles) nAb against IFN-α (5 µg/ml) were prepared at the indicated time points (24, 48, and 72 hrs post-infection with SeV). *D*, HCV RNA copies in HuS-E/2 cells pre-treated with (open circles) and without (closed circles) IFN-α nAb at the indicated time points (24, 48, 72, and 96 hrs post-infection with HCVcc) were measured by qRT-PCR. *E,* Time course expression of IFN-α1, IFN-β, IFN-λ1, IFN-λ3, RIG-I and IRF-7 mRNAs in the cells pre-treated with (open circles) or without (closed circles) IFN-α nAb during early stage of HCV infection (6, 12, 18, and 24 hrs post-infection with HCVcc). For each analysis, the results are normalized to the value obtained from the mock treatment. Error bars represent the calculated SD from the results obtained in three independent experiments (*B, C, D, E*).

To examine whether IFN-α produced in the cells without virus infection actually plays a role in prevention of viral infection, the permissiveness of HuS-E/2 cells, which were pretreated with and without IFNAR2 nAb, against SeV infection was investigated. Compared to the cells without pretreatment of nAb (shown as control panels in Fig. 6*A*), the number of SeV infected cells (shown in red) was increased in the cells with treatment both 6 and 9 hrs after infection in time-dependent manner (Fig. 6*B*). We also assessed the proliferation of SeV in HuS-E/2 cells with or without pretreatment of IFN-α nAb by quantitative estimation of SeV genomic RNA. As shown in Fig. 6*C*, the increase of SeV genomic RNA levels was clearly observed in the cells with the pre-treatment at 12 hrs post-infection, suggesting that the preexisting IFN-α play a suppressive role on SeV proliferation during early phase of infection. In addition, to investigate the effect of constitutive IFN-α on the hepatotropic virus, we examined the infection and proliferation of HCVcc in HuS-E/2 cells with or without pretreatment of IFN-α nAb. As shown in Fig. 6*D*, the transient infection and proliferation of HCVcc was observed in the HuS-E/2 cells as previously reported [10]. It was clearly observed that HCV RNA levels in the cells with the pretreatment were significantly higher than the cells without pretreatment from 24 to 48 hrs post-infection, although the significant difference was not observed in each cells from 72 to 96 hrs post-infection (Fig. 6*D*). Next, the expression of several IFN related genes in HuS-E/2 cells with or without the pretreatment was examined after HCVcc infection. As shown in Fig. 6*E*, compared to the cells without pretreatment, the delayed increases of RNAs for IFN-α1, IFN-β, IFN-λ1, IFN-λ3, RIG-I and IRF-7 after HCV infection were observed in HuS-E/2 cells with the pretreatment, although induction patterns were varied among those genes. These results suggested that IFN-α produced from HuS-E/2 cells without virus infection contributes to rapid antiviral innate immune response of the cells to limit the proliferation of HCV during the initial stage of infection.

## Discussion

In this study, we showed that IFN-α1 gene is expressed in HuS-E/2 cells without virus infection as in the case of PHH, albeit at a low level and that the IFN-α, including IFN-α1, functions to elevate the expression of the genes related with anti-viral innate immune system in the cells. Previously, the expression pattern of the genes related with innate immunity of the HuS-E/2 cells, was shown to be similar to that of PHH [10]. The constitutive expression of IFN-α1 gene was also previously observed in human liver tissue [8]. Our results, therefore, suggested that the previous detection of IFN-α1 mRNA in normal human liver is due in part at least to the gene expression in hepatocytes in that tissue. The constitutive production of type I IFN has been reported previously in several tissues mainly concerning IFN-β [17], [18], [19], [20]. The mechanisms that support the constitutive production of IFN-β have been relatively studied well and revealed to be involved with multiple transcription factors, such as c-Jun and RelA [6]. However, molecular mechanism that controls the steady-state production of IFN-α has been largely unclear. The transcriptional promoter region of IFN-α1 gene contains two regulatory elements, one is homologous to the positive regulatory domain I (PRDI) of the IFN-β gene promoter [21], [22], [23] and another is virus-responsive enhancer module, as proposed to a TG-like domain [24], [25]. Our previous report showed that IRF-7 gene is constitutively expressed in HuS-E/2 cells [10]. IRF-7, together with IRF-3, is known to play a pivotal role in the induction of IFN-α and IFN-β genes through binding with PRDI in cells infected with virus [3], [4], although it was suggested that IRF-7, rather than IRF-3, is important to suppress the infection and replication of HCV in the cells [10]. The basal expression of IFN-α and IFN-β genes, however, was reported not to depend on these regulatory factors [26]. We also observed that silencing IRF-7 in HuS-E/2 cells with shRNA method did not diminish the steady-state level expression of IFN-α1 (data not shown). We found some sequences homologous to the binding sites for some hepatocyte-specific transcription factors, including hepatocyte nuclear factor 1α (HNF1α), HNF1β, and HNF4α within the region 5000 base pairs upstream of the transcription start site of IFN-α1 gene using computational promoter analysis (data not shown). It, therefore, may be possible that the tissue-specific transcription factor contributes to the expression of IFN-α1 gene in a tissue-specific manner. As a recent study showed that tissue–specific differences in IFN genes or ISG expression can be attributed in part to the epigenetic regulation [27], it is probable that the innate immune phenotypes of IFN-α gene in human hepatocytes is also associated with tissue-specific patterns of histone modification. Further study is needed to clarify the molecular mechanisms of constitutive expression of IFN-α1 gene in human hepatocytes.

The results obtained from this study showed the functional role of the constitutive IFN-α in human hepatocytes on the immediate innate immune response against RNA virus infection, including HCV, through augmentation of the steady-state level expression of several genes related to detection of the infection and induction of IFN systems, such as RIG-I, IRF-7, and IFNs genes. Rapid activation of IFN system should be important to suppress the expansion of viral infection. The constitutive IFN-β has been reported previously in several tissues and was demonstrated to strengthen IFN response toward viral infection [17], [18], [19], [20]. This constitutive IFN-β involves a positive feedback loop as proposed in a “revving-up model” in such tissues [28]. The weak cellular signals constantly introduced by constitutive IFN-β allows cells to elicit a more robust response against viral infection than the cells without such signals [17]. This signaling likely occasion induction of the IRF-7 gene without viral infection as a priming effect [29]. Cardiac myocytes was reported to produce higher basal IRF-7 without viral infection through the Jak-STAT pathway activated with preexisting IFN-β for instant antiviral response [20]. Plasmacytoid dendritic cells (pDCs) are known to produce IFN-α and IRF-7 constitutively just like human hepatocytes reported in this study. pDCs respond rapidly and effectively to a range of viral pathogens with high production of IFN-α in constitutive IRF-7 production dependent manner [30], [31], [32]. These suggested that IRF7, of which gene expression is induced by constitutive type-I interferon, both IFN-α and IFN-β, in those cells, plays a crucial role in the priming effect on the consecutive and rapid anti-viral innate immune response.

The liver is the largest solid organ in the body with dual inputs for its blood supply. It receives 80% of its blood supply from the gut through the portal vein, which is rich in bacterial products, environment toxins, and food borne pathogens. The remaining 20% of the blood is supplied from vascularization by the hepatic artery [33]. This high exposure to pathogens may require that the liver has an efficient and rapid defensive mechanism against possible frequent infection. Although most pathogens that get at the liver are killed by local innate and adaptive immune responses, hepatitis viruses (such as HBV and HCV) which gain the ingenious function to escape immune control persist in hepatocytes [34], [35], [36], [37], [38]. Therefore, further study to reveal the role of steady-state production of IFN-α1 in human hepatocytes may provide new insights into the virus-cell interaction and chronic infection of hepatotropic viruses.

In addition to the importance for the antiviral effect, it has been also proposed that the constitutive IFN-β primes for an efficient subsequent response to other cytokines and are also important for immune homeostasis [39], [40], [41], maintenance of bone density [42] and antitumor immunity [43]. Further analysis of the possible role of constitutive IFN-α on the other physiological events in human hepatocytes may be required.

## Supporting Information

Table S1 **List of names and sequences of the primers and expected sizes of RT-PCR products using those primers.**
(TIF)
