# Supplementary figures and images for: Critical Role of Interferon-α Constitutively Produced in Human Hepatocytes in Response to RNA Virus Infection
Source: PLoS One. 2014 Feb 26;9(2):e89869. doi: 10.1371/journal.pone.0089869 (PMC3935935; doi:10.1371/journal.pone.0089869)

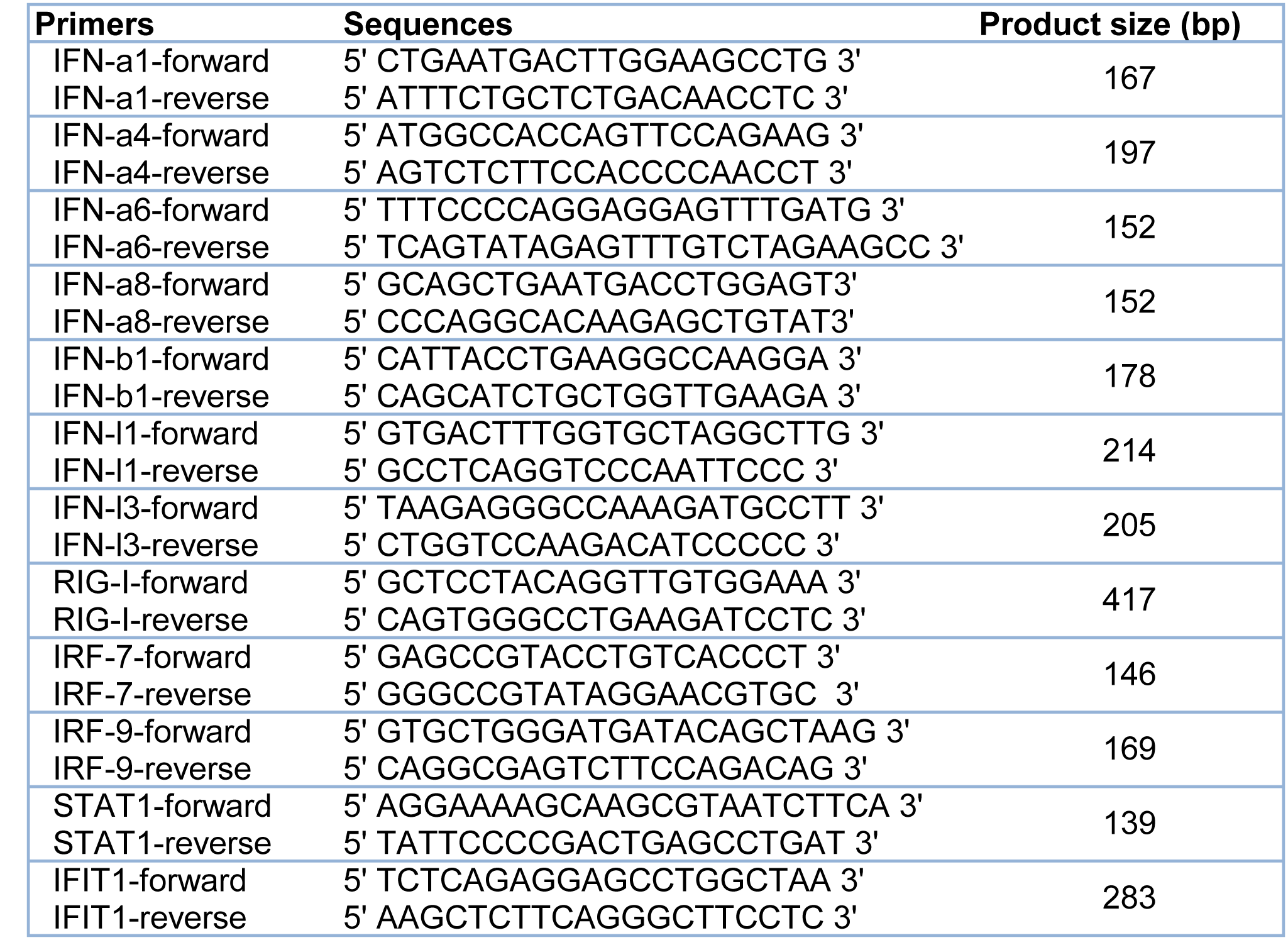

Supplement: Table S1 — List of names and sequences of the primers and expected sizes of RT-PCR products using those primers. (TIF) [file pone.0089869.s001.tif]
